# Dieckol, a Major Marine Polyphenol, Enhances Non-Rapid Eye Movement Sleep in Mice *via* the GABA_A_-Benzodiazepine Receptor

**DOI:** 10.3389/fphar.2020.00494

**Published:** 2020-04-17

**Authors:** Minseok Yoon, Jin-Soo Kim, Sangwoo Seo, Kiwon Lee, Min Young Um, Jaekwang Lee, Jonghoon Jung, Suengmok Cho

**Affiliations:** ^1^ Korea Food Research Institute, Wanju-gun, South Korea; ^2^ Department of Seafood Science and Technology, Institute of Marine Industry, Gyeongsang National University, Tongyeong, South Korea; ^3^ WCU Biomodulation Major, Department of Agricultural Biotechnology, Seoul National University, Seoul, South Korea; ^4^ Advanced Institutes of Convergence Technology, Seoul National University, Suwon, South Korea; ^5^ Department of Food Science and Technology/Institute of Food Science, Pukyong National University, Busan, South Korea

**Keywords:** dieckol, phlorotannins, marine polyphenols, sleep, electroencephalogram, hypnotic

## Abstract

We had previously demonstrated that phlorotannins, which are marine polyphenols, enhance sleep in mice *via* the GABA_A_-benzodiazepine (BZD) receptor. Among the constituents of phlorotannin, dieckol is a major marine polyphenol from the brown alga *Ecklonia cava*. Although phlorotannins are known to exert hypnotic effects, the sleep-enhancing effect of dieckol has not yet been determined. We evaluated the effect of dieckol on sleep-wake state of mice by analyzing electroencephalograms (EEGs) and electromyograms. Flumazenil, a GABA_A_-BZD antagonist, was used to investigate the molecular mechanism underlying the effects of dieckol on sleep. The polygraphic recordings and corresponding hypnograms revealed that dieckol accelerated the initiation of non-rapid eye movement sleep (NREMS); it shortened sleep latency and increased NREMS duration. According to the change in time-course, dieckol showed sleep-enhancing effects by increasing the amount of NREMS and decreasing wakefulness during the same hours. Additionally, sleep quality was evaluated by analyzing the EEG power density, and dieckol was found to not affect sleep intensity while zolpidem was found to reduce it. Finally, we treated mice with zolpidem or dieckol in combination with flumazenil and found the latter to inhibit the sleep-enhancing effect of dieckol and zolpidem, thereby indicating that dieckol exerts sleep-enhancing effects by activating the GABA_A_-BZD receptor, similar to zolpidem. These results implied that dieckol can be used as a promising herbal sleep aid with minimal side effects, unlike the existing hypnotics.

## Introduction

Insomnia is a highly prevalent complaint in modern society (Jespersen et al., 2015). Sleep deprivation exerts negative effects on physical and mental performance, mood, as well as the immune system; overall, it affects the quality of life (Alami et al., 2018). With insomnia becoming increasingly prevalent, herbal sleep aids are gaining popularity worldwide as alternatives to prescription drugs for improving sleep quality or treating insomnia (Meletis and Zabriskie, 2008). Therefore, the sleep-enhancing effects of herbal plants or phytochemicals have been reported widely.

GABA_A_ receptors have been considered to be important molecular targets for the development of sleep-enhancing drugs and herbal sleep aids. In fact, polyphenols, mainly flavonoids, exert their hypnotic effects through the positive allosteric modulation of GABA_A_ receptors (Johnston, 2015). For example, honokiol and magnolol increase non-rapid eye movement sleep (NREMS) in mice by acting on the GABA_A_-benzodiazepine (BZD) receptor (Chen et al., 2012; Qu et al., 2012). Glabrol, which is licorice component, has been characterized as a GABA_A_-BZD receptor ligand that exhibits hypnotic effects (Cho et al., 2012a). Although many studies have been conducted to reveal the hypnotic effects of polyphenols, they have all been limited to terrestrial plants (Cho and Shimizu, 2015). Recently, we reported for the first time that phlorotannins, which are marine polyphenols, enhance sleep in mice *via* the GABA_A_-BZD receptor (Cho et al., 2014). Phlorotannins consist of oligomers and polymers of phloroglucinol as the basic unit (Koivikko et al., 2007). Currently, approximately 150 phlorotannin compounds, such as dieckol, bieckol, eckol, triphlorethol A, and trifucol, have been identified from various brown seaweeds (Kim et al., 2014). Among the constituents of phlorotannin, dieckol is regarded as an indicator of phlorotannin extracts from brown alga based on the amount of it present (Shibata et al., 2002; Shibata et al., 2004; Cho et al., 2014). Although hypnotic effects of phlorotannins have been reported, the effect of dieckol on sleep remains to be investigated.

In this study, we aimed to investigate whether dieckol indeed exhibits sleep-enhancing effects by analyzing its effects on the sleep-wake profiles of C57BL/6N mice using recorded electroencephalograms (EEGs) and electromyograms (EMGs). In addition, the underlying GABAergic mechanism of dieckol was delineated using flumazenil, an antagonist of the GABA_A_-BZD receptors.

## Materials and Methods

### Isolation of Dieckol From Phlorotannin Preparation

**Figure 1:**
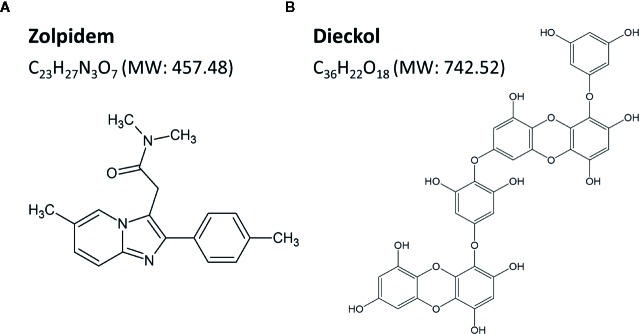
Chemical structures and molecular weights (MW) of zolpidem **(A)** and dieckol **(B)**.

To obtain highly pure phlorotannin preparation (PRT), the ethanol extract of *Ecklonia cava* was purified using a Diaion HP-20 resin (Mitsubishi Chemical Industries Ltd., Tokyo, Japan) (Cho et al., 2014). The total content of phlorotannin was 900 mg of phloroglucinol equivalents/g of dry extract, as determined by the Folin-Ciocalteu method, and standardized to 67 mg/g dry extract of dieckol. Next, PRT (250 g) was partitioned by ethyl acetate (EA, 110 g) and H_2_O (132 g). The EA fraction was subjected to SiO_2_ (Kiesel gel 60, Merck, Darmstadt, Germany) column chromatography (CC). The column was eluted using mixtures of CHCl_3_-methanol (MeOH) at a ratio of 10:1, 6:1, 3:1, and 1:1 in sequence, and the eluates were collected into five sub-fractions (E_1_ – E_5_) by thin-layer chromatography. The sub-fraction E_4_ (68 g) was further separated with Sephadex LH-20 CC (Ø 2.5 cm × 50 cm, 80% MeOH) to get four sub-fractions (E_4-1_–E_4-4_), from which the sub-fraction E_4-3_ (850 mg) was subjected to ODS-C_18_ reverse chromatography. Finally, highly pure dieckol (87.2%) (**Figure 1B**) was obtained by Sephadex LH-20 CC.

### Animals and Treatment

Eleven-week-old C57BL/6N male mice (weighing 25–28 g) were obtained from Koatech Animal Inc. (Pyeongtaek, Korea) and acclimatized for a week before being used in experiments. The animals were randomly assigned to different experimental groups. They were housed in individual cages and fed sterilized food and water. The cages were placed in an insulated sound-proof recording room with automatically controlled light/dark cycle (12-h/12-h, respectively; lights on at 05:00, illumination intensity of approximately 300 lux). Ambient temperature and relative humidity were maintained at 23 ± 0.5°C and 55 ± 2%, respectively.

Dieckol was freshly dissolved in sterile saline including 2% dimethyl sulfoxide (DMSO) and 0.5% carboxymethyl cellulose (CMC) before use. Mice were divided into three groups (n = 6–7 per group), and were orally administered (p.o.) dieckol (50, 100, or 150 mg/kg) at 17:00 on the day of the experiment. Zolpidem (Ministry of Food and Drug Safety, Cheongwon-gun, Chungcheongbuk-do, Korea), a potent GABA_A_-BZD agonist, was selected as the positive control (**Figure 1A**). Flumazenil (Sigma-Aldrich Inc., St. Louis, MO, USA) was dissolved in sterile saline for intraperitoneal injection 15 min prior to oral administration of vehicle, dieckol or zolpidem. For baseline data, mice were treated with the vehicle at 17:00 (p.o.).

### Vigilance State Analysis Based on Polygraphic Recordings

**Figure 2:**
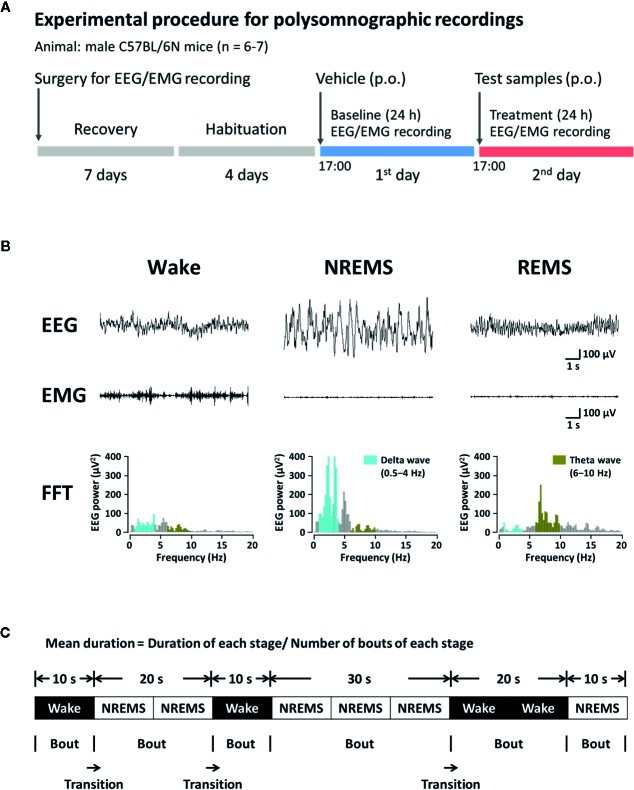
Experimental procedure **(A)**, typical EEG, EMG, and FFT spectra **(B)**, and definition of sleep-wake episodes **(C)** in C57BL/6N mice. EEG, electroencephalogram; EMG, electromyogram; FFT, fast Fourier transform; NREMS, non-rapid eye movement sleep; REMS, rapid eye movement sleep; Wake, wakefulness.

**Figure 2A** shows the sleep analysis with respect to the experimental procedure and timeline. To record polygraphic signals, a head mount (#8201; Pinnacle Technology, Inc., Lawrence, KS, USA) equipped with EEG and EMG electrodes was implanted in mice under pentobarbital anesthesia (50 mg/kg, i.p.) (Cho et al., 2014). The head and neck of anesthetized mouse were shaved and cleaned with 70% alcohol before surgery. The anesthetized mouse was incised and the front edge of head mount was inserted 3 mm anterior to the bregma on skull. Four stainless-steel screws for EEG recording were inserted into the skull. Two EMG wire electrodes were placed bilaterally into the nuchal muscles. Dental cement was used to fix the head mount on the skull. Once surgery was completed, the mice were moved to separate cages for recovery at least for a week. Three to four days before the experiments, animals were adapted to the recording conditions. EEG and EMG recordings were performed under freely moving condition using a slip ring. The EEG and EMG signals were collected using the PAL-8200 data acquisition system (Pinnacle Technology, Inc.). All signals were amplified (100×), filtered (high-pass filter: 0.5 Hz for EEG and antialiasing filter: 10 Hz for EMG), and stored at a sampling rate of 200 Hz. Sleep-wake cycle was monitored over 48 h, including baseline data acquisition and experimentation. Recording of baseline was conducted over 24 h in each mouse, beginning at 17:00. Obtained baseline from each mouse was used as the control. Mice were considered to be falling asleep when there was no detectable signal in EMG. The vigilance states were automatically classified by a 10-s epoch as wakefulness (Wake), rapid eye movement sleep (REMS), or non-REM sleep (NREMS) using SleepSign version 3.0 (Kissei Comtec, Nagano, Japan) according to the standard criteria (Kohtoh et al., 2008). At the end of step, visual examination was done to define sleep-wake stages and to correct, if necessary. Sleep latency was measured by the time taken for the first NREMS episode to appear (lasting for at least 120 s) from the time of drug administration. Delta activity (the range of 0.5–4 Hz) during NREMS was first averaged across the individual animal and then they were summed up, and subsequently normalized to obtain the percentage of corresponding mean delta activity during NREMS. **Figure 2B** shows the typical waveforms of EEG and EMG, and the fast Fourier transform spectra of delta and theta waves. Bouts of each stage were defined as the periods of one or more consecutive 10-s epochs (**Figure 2C**).

### Statistical Analysis

Data plotted in figures are indicated as the mean ± SEM. Statistical analysis was performed with GraphPad Prism 5.0 software (GraphPad Software Inc., San Diego, CA, USA). Sleep latency, amount of NREMS and REMS, mean duration, stage transition number, and the number and duration of bouts were analyzed by the paired Student’s *t*-test. For time-course of the hourly amounts of each stage and delta activity were assessed by two-way ANOVA with Bonferroni post-test. *P*-values less than 0.05 were considered significant for all statistical tests.

## Results

### Effect of Dieckol on Sleep Latency and Amounts of REMS and NREMS in C57BL/6N Mice

**Figure 3:**
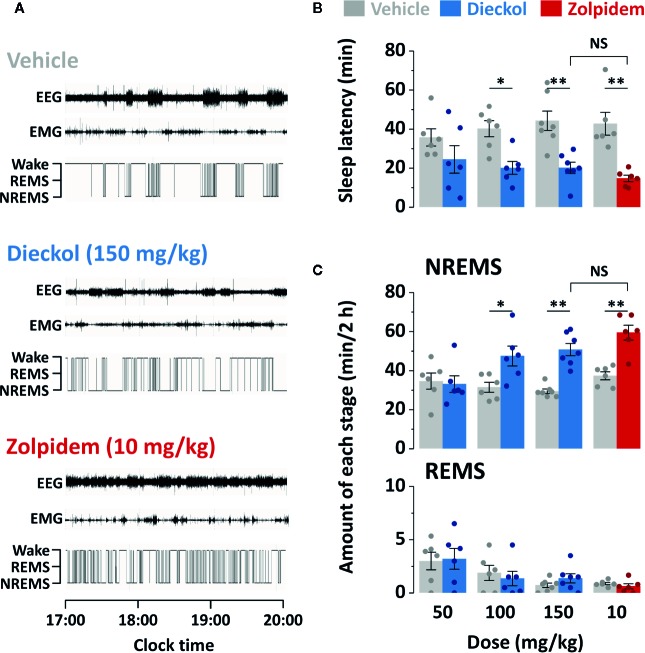
Effect of dieckol and zolpidem on sleep-wake profiles in C57BL/6N mice. **(A)** Representative EEG and EMG signals, and corresponding hypnograms in a mouse treated with dieckol or zolpidem. **(B)** Effects of dieckol and zolpidem on sleep latency. **(C)** Amounts of NREMS and REMS during the 2 h period after administration of dieckol or zolpidem. Gray bars indicate the baseline day (vehicle administration). Each value represents the mean ± SEM of each group (n = 6–7) with data points. *p < 0.05 and **p < 0.01, significant difference compared to the vehicle (paired Student’s *t*-test). EEG, electroencephalogram; EMG, electromyogram; NREMS, non-rapid eye movement sleep; NS, no significance; REMS, rapid eye movement sleep; Wake, wakefulness.

To investigate the effect of orally administered dieckol on sleep structure, we analyzed the sleep architecture in C57BL/6N mice using EEG and EMG recordings. **Figure 3A** shows a representative example of polygraphic recordings and corresponding hypnograms from a single mouse during the first 3 h after treatment with vehicle, dieckol, and zolpidem. Values of sleep latency were 19.2 ± 3.2 min in mice administrated 150 mg/kg dieckol and 14.8 ± 1.7 min in those administered 10 mg/kg zolpidem (**Figure 3B**). Sleep latency with dieckol and zolpidem was significantly shorter than that after vehicle treatment, specifically 44.5 ± 5.9 and 42.8 ± 5.8 min, respectively. Dieckol induced short sleep latency in mice, indicating that it accelerated the initiation of NREMS, similar to zolpidem.

To compare the effect of dieckol and zolpidem on sleep duration, we computed the total amounts of NREMS and REMS for 2 h after the administration of both chemicals (**Figure 3C**). As expected, the zolpidem-administered group, as a positive control, showed significantly (*p* < 0.01) increased total NREMS (by 1.6-fold) compared to that in the vehicle-treated group. Dieckol administration increased NREMS duration in a dose-dependent manner. Dieckol concentrations of 100 and 150 mg/kg significantly increased NREMS by 1.4-fold (*p* < 0.05) and 1.7-fold (*p* < 0.01), respectively, as compared to the vehicle. In particular, there was no significant differences in sleep latency and NREMS amount between 150 mg/kg dieckol and 10 mg/kg zolpidem group. However, dieckol at 50 mg/kg did not affect either sleep latency or NREMS amount. In addition, changes in REMS amount were not found after dieckol or zolpidem administration, as compared to vehicle.

### Effects of Dieckol on Time-Course Changes of Sleep-Wake Stage in C57BL/6N Mice

**Figure 4:**
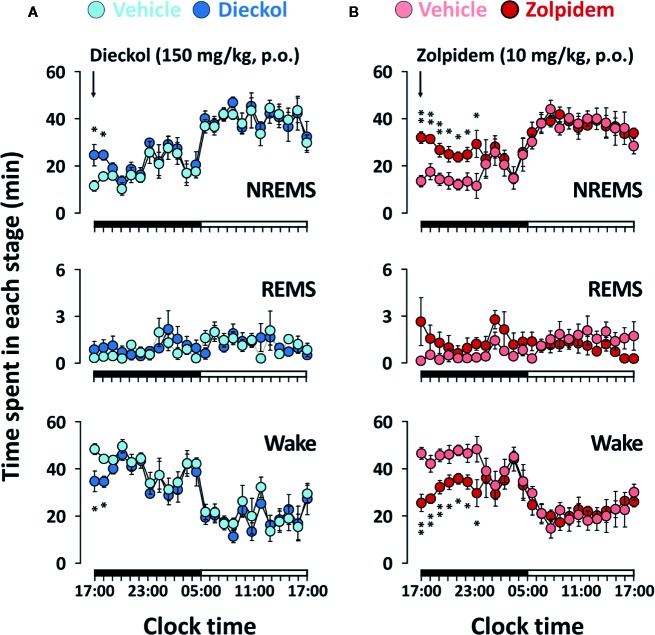
Effects of dieckol **(A)** and zolpidem **(B)** on time-course changes in NREMS, REMS, and Wake for 24 h in C57BL/6N mice. Light and dark circles indicate the baseline day (vehicle) and experimental day (dieckol or zolpidem), respectively. Each circle represents the hourly mean ± SEM (n = 6–7) of NREMS, REMS, and Wake. *p < 0.05 and **p < 0.01, significant difference compared to the vehicle (two-way ANOVA with Bonferroni post-test). NREMS, non-rapid eye movement sleep; REMS, rapid eye movement sleep; Wake, wakefulness.

**Figure 4** shows the time-course changes of the hourly amounts of NREMS, REMS, and Wake. Dieckol (150 mg/kg) showed a 2.1- and 1.30-fold increase in the amount of NREMS during the first and second hours, respectively, relative to the vehicle (**Figure 4A**). This improvement in NREMS was accompanied by reduction in Wake during the same hours. After an initial increase in NREMS, sleep architecture did not change significantly during the subsequent periods. Moreover, dieckol at 100 mg/kg exhibited similar time-course profiles, although the enhancing effect of sleep was minor, lasting over approximately 2 h after administration (data not shown). Unlike dieckol, zolpidem at 10 mg/kg significantly enhanced NREMS by 7 h (**Figure 4B**). Neither of dieckol and zolpidem was significantly different compared to each vehicle over 24 h.

### Effects of Dieckol on Sleep-Wake Episode and Delta Activity

**Figure 5:**
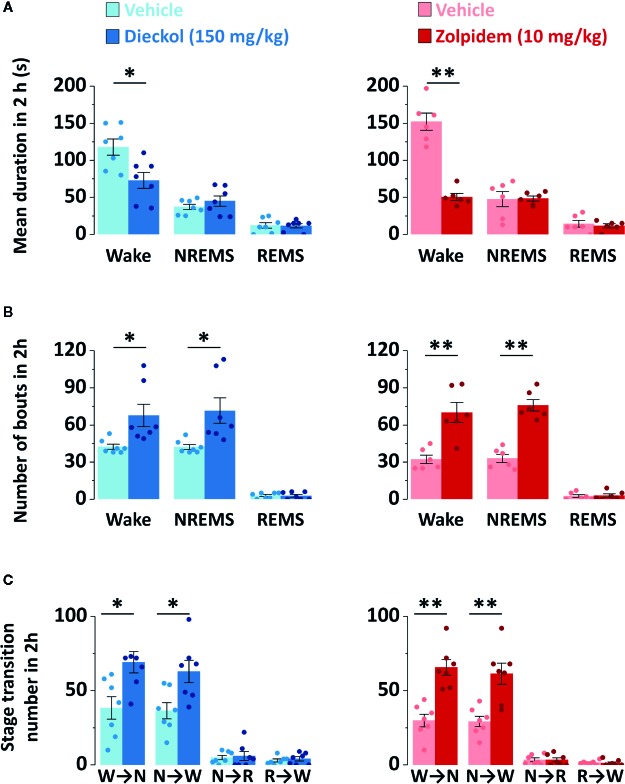
Characteristics of sleep-wake episodes in C57BL/6N mice after oral administration of dieckol and zolpidem. **(A)** Mean duration and **(B)** total number of NREMS, REMS, and Wake bouts in a 2 h period after the administration of dieckol and zolpidem. **(C)** Sleep-wake state transitions during the 2 h following the administration of dieckol and zolpidem. Light and dark bars indicate the baseline day (vehicle administration) and experimental day (dieckol or zolpidem administration), respectively. Each column represents the mean ± SEM (n = 6–7) with data points. *p < 0.05 and **p < 0.01, significant difference compared to the vehicle (paired Student’s *t*-test). EEG, electroencephalogram; EMG, electromyogram; NREMS (or N), non-rapid eye movement sleep; REMS (or R), rapid eye movement sleep; Wake (or W), wakefulness.

**Figure 6:**
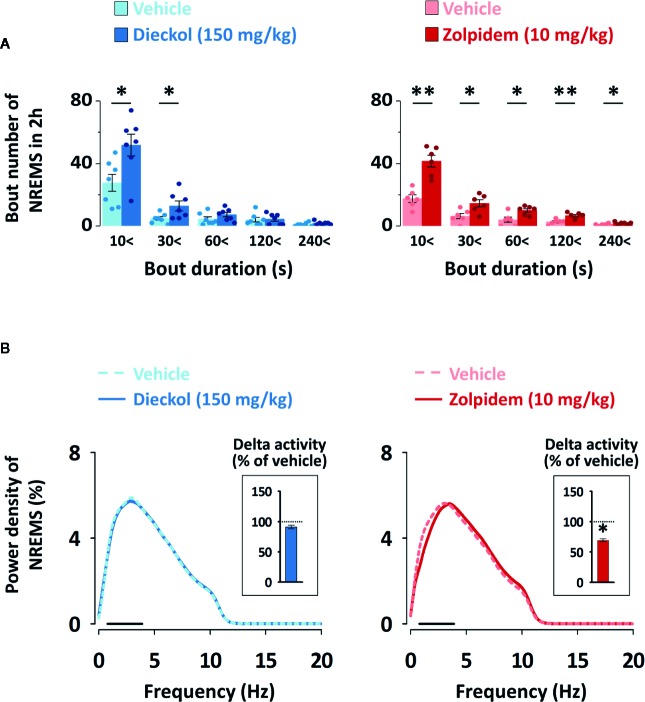
**(A)** Changes in the number of NREMS and Wake bouts of different durations in C57BL/6N mice after oral administration of dieckol or zolpidem. Light and dark bars indicate the baseline day (vehicle administration) and experimental day (dieckol or zolpidem administration), respectively. Each column represents the mean ± SEM (n = 6–7) with data points. *p < 0.05 and **p < 0.01, significant difference compared to the vehicle (paired Student’s *t*-test). **(B)** Electroencephalogram (EEG) power density curves of NREMS caused by dieckol and zolpidem. Delta activity, an index of sleep intensity, is shown in the inset histogram. The dash (▬) represents the range of the delta wave (0.5‒4 Hz). *p < 0.05, significant difference compared to the vehicle (two-way ANOVA with Bonferroni post-test). NREMS, non-rapid eye movement sleep.

To evaluate the sleep-enhancing effect of dieckol, mean duration and total number of NREMS, REMS, and Wake episodes were analyzed. Dieckol (150 mg/kg) and zolpidem (10 mg/kg) significantly decreased the mean duration of Wake by 53.8% (*p * < 0.01) and 38.0% (*p* < 0.05), respectively, without affecting the mean duration of NREMS or REMS (**Figure 5A**). Moreover, both dieckol and zolpidem produced an increase in the number of NREMS bouts by 1.7- and 2.2-fold, and those of Wake bouts by 1.6- and 2.1-fold, respectively (**Figure 5B**). REMS bouts remained unchanged. In addition, both dieckol and zolpidem showed an increase in the number of state transitions from Wake to NREMS and NREMS to Wake (**Figure 5C**). However, we did not find any change in the number of transitions from NREMS to REMS or from REMS to Wake. Finally, dieckol induced an increase in bout number for NREMS, ranging from 10 to 60 s each. Zolpidem also raised the number of bouts of short-length NREMS; it resulted in significant differences in middle-length NREMS bouts, ranging from 60 to 480 s (**Figure 6A**).

In order to evaluate the sleep quality, delta activity was analyzed from the EEG power density during NREMS. As shown in **Figure 6B**, there was no significant difference in EEG power density (0–20 Hz), and delta activity (frequency range of 0.5–4 Hz), for NREMS between the dieckol- and vehicle-treated mice. However, zolpidem significantly (*p* < 0.05) decreased the delta activity. These results suggested that, unlike zolpidem, dieckol increased sleep quantity without loss of sleep intensity.

### Possible Mechanism of Action of Dieckol

**Figure 7:**
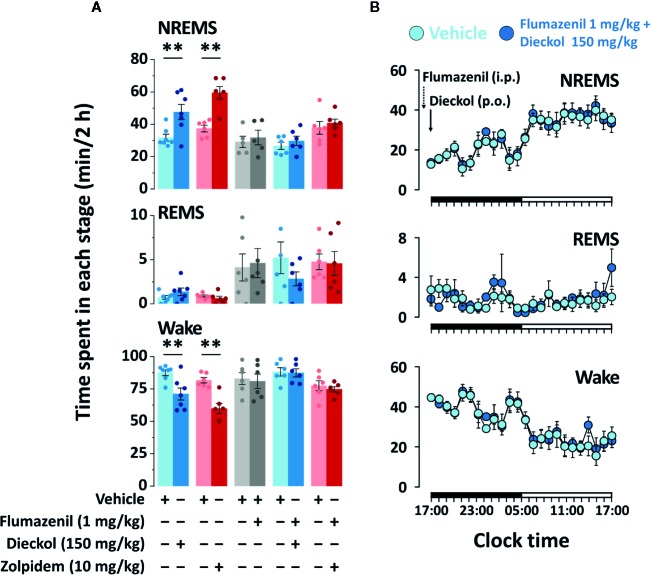
Effect of flumazenil treatment on dieckol- and zolpidem-induced sleep in C57BL/6N mice. **(A)** Amount of NREMS, REMS, and Wake for 2 h after pretreatment with flumazenil (1 mg/kg, i.p. at 16:45 h) and oral administration of dieckol (150 mg/kg, at 17:00 h) or zolpidem (10 mg/kg, at 17:00 h) and each vehicle in mice. Each column represents the mean ± SEM (n = 6-7) with data points. **p < 0.01, significant difference compared to the vehicle (paired Student’s t-test). **(B)** Time-course changes in NREMS, REMS, and Wake after administration of vehicle, flumazenil, and dieckol. The horizontal filled and open bars on the X-axis (time) indicate the 12 h dark and 12 h light periods, respectively.

In our previous study, we had suggested that phlorotannin extracts, with dieckol as the major component, enhance NREMS through the activation of BZD binding site of GABA_A_ receptor (Cho et al., 2014). This led us to investigate the sleep-enhancing effect of dieckol, if at all, through the GABAergic system. To confirm the action of dieckol on GABA_A_-BZD receptor, mice were pretreated with flumazenil, which is the specific GABA_A_-BZD receptor antagonist. There was no significant difference in sleep architecture of mice with the flumazenil (1 mg/kg) injection alone (**Figure 7A**). Effects of zolpidem, the GABA_A_-BZD agonist, were completely inhibited by flumazenil. Flumazenil also fully suppressed the sleep-enhancing effect of dieckol. The time-course changes in each stage over a period of 24 h revealed that dieckol did not affect sleep architecture in presence of flumazenil, in comparison with the vehicle (**Figure 7B**). These results implied that the sleep-enhancing effects of dieckol could be *via* the modulation of GABA_A_ receptor at the BZD binding site.

## Discussion

In this study, we found both dieckol and zolpidem to reduce sleep latency and increase NREMS without altering REMS. Moreover, we showed the amounts of NREMS between zolpidem at 10 mg/kg and dieckol at 150 mg/kg to not be significantly different (*p* > 0.05). Dieckol exerted a significant effect during the first 2 h after administration. During the subsequent period, we did not observe further significant disruption of sleep architecture. These results suggested that dieckol induces NREMS without causing harmful effects after sleep induction (Masaki et al., 2012). Considering the finding that dieckol reduced the mean duration of Wake episodes and increased the total number of NREMS bouts, we could strongly suggest that maintenance of Wake was inhibited by dieckol (Masaki et al., 2012). Delta activity is a marker of the depth or intensity of NREMS (Winsky-Sommerer, 2009). The main effects of BZD agents have been reported as shortening of sleep latency and enhancement of sleep duration while suppressing delta activity (Lancel et al., 1997; Tobler et al., 2001; Kopp et al., 2004a). Zolpidem has been reported to produce a decrease in delta activity during NREMS (Kopp et al., 2004b; van Lier et al., 2004; Alexandre et al., 2008). Similarly, we also found delta activity to be significantly decreased by zolpidem; however, it remained unchanged by dieckol. Our results showed that dieckol produces NREMS similar to physiological sleep (Xu et al., 2014). Classical BZD-hypnotics have high affinity for α_1_, α_2_, α_3_, and α_5_ subunits of GABA_A_ receptors (Barnard et al., 1998; Möhler et al., 2002). Zolpidem has been reported to have a high affinity to α_1_-GABA_A_ receptors, and an intermediate affinity to α_2_- and α_3_-GABA_A_ receptors. However, it has a limitation in binding to α_5_-GABA_A_ receptors (Crestani et al., 2000). Although zolpidem suppresses delta activity by binding to α_2_ subunits of GABA_A_ receptors, as does diazepam (Kopp et al., 2004a), the hypnotic action of zolpidem is mediated by GABA_A_ receptors containing α_1_ subtypes (Sanna et al., 2002; Sanger, 2004; Tsai et al., 2013). Therefore, we suggest that the action of dieckol and zolpidem is mediated by different GABA_A_ receptor subunits.

We had previously reported that dieckol works as a ligand of GABA_A_-BZD receptor and its K*_i_* (binding affinity) value for [^3^H]-flumazenil binding is 3.072 µM (Cho et al., 2012b). These results suggested that the hypnotic effect of dieckol is attributed to GABAergic pathways. For this reason, we examined the effect of dieckol with co-application of flumazenil, which is a specific GABA_A_-BZD receptor antagonist, on the sleep-enhancing effect of dieckol *in vivo*. Flumazenil acts as an inhibitor of the hypnotic effect of GABA_A_-BZD receptor agonists (such as zolpidem) by interfering with their binding sites (Johnston, 2005). In the current study, we found the sleep-enhancing effect of dieckol, similar to that of zolpidem, to be completely blocked by flumazenil compared to that in the vehicle. Previously, honokiol and magnolol had been reported to promote NREMS by activating the BZD binding site of GABA_A_ receptor, since their somnogenic effects and activation of ventrolateral preoptic area neurons were blocked by flumazenil (Chen et al., 2012; Qu et al., 2012). These findings supported our hypothesis that dieckol enhances sleep by acting as a positive allosteric modulator of GABA_A_ receptors at the BZD binding site, similar to zolpidem.

## Conclusion

We demonstrated the sleep-enhancing effects of dieckol in mice and established that these effects are mediated *via* the GABA_A_-BZD receptor. Our results provide important insights that can contribute to the development of hypnotics with new structures, since dieckol, derived from brown seaweed, has a different structure compared to the polyphenols of terrestrial plants and other hypnotic drugs. However, for the development of novel polyphenol-based hypnotics, further electrophysiological studies on the *in-vivo* effects of chronic administration would be recommended.

## Data Availability Statement

The raw data supporting the conclusions of this manuscript will be made available by the authors, without undue reservation, to any qualified researcher.

## Ethics Statement

All procedures involving animals were conducted in accordance with the animal care and use guidelines of the Korea Food Research Institutional Animal Care and Use Committee (permission number: KFRI-M-12027).

## Author Contributions

MY, J-SK, and SC conceived and designed the experiments. MY, MU, JL, and JJ performed the experiments. Data analysis was performed by MY and SC. The manuscript was prepared by MY, SS, and SC. KL contributed expert opinion to the manuscript correction.

## Funding

This research was supported by the Main Research Program (E0164501-04 and E0164503-02) of the Korea Food Research Institute (KFRI), funded by the Ministry of Science and ICT.

## Conflict of Interest

The authors declare that the research was conducted in the absence of any commercial or financial relationships that could be construed as a potential conflict of interest.
